# Unveiling the Small Molecules Binding Site of CD36 Cell Surface Receptor Through Docking and Molecular Dynamics Simulations

**DOI:** 10.2174/0109298673413172251202110617

**Published:** 2026-02-26

**Authors:** Naomi Montes-Mondragón, Rosa Salgado-Brito, José-Rubén García-Sánchez, Martiniano Bello, José Correa-Basurto, Vicente Sandoval-Herrera, Sergio-Roberto Zamudio-Hernández, David Méndez-Luna

**Affiliations:** 1 Universidad Simón Bolívar, Av. Río Mixcoac Nº 48, Col. Insurgentes, Mixcoac, Benito Juárez, Ciudad de México C.P. 03920, México;; 2 Laboratorio de Oncología Molecular y Estrés Oxidativo de la Escuela Superior de Medicina, Instituto Politécnico Nacional, Plan de San Luis y Díaz Mirón, s/n, Col. Casco de Santo Tomas, Ciudad de México, 11340, México;; 3 Laboratorio de Diseño y Desarrollo de Nuevos Fármacos e Innovación Biotecnológica, Escuela Superior de Medicina, Instituto Politécnico Nacional, Plan de San Luis y Díaz Mirón s/n, Col. Casco de Santo Tomas, Alcaldía Miguel Hidalgo, C.P. 11340, Mexico City, México;; 4 Departamento de Fisiología, Escuela Nacional de Ciencias Biológicas, Instituto Politécnico Nacional, Zacatenco, Av. Wilfrido Massieu 399, Col. Nueva Industrial Vallejo, Alcaldía Gustavo A. Madero, C.P. 07738, Ciudad de México, México

**Keywords:** CD36, docking and MD simulations, small-molecules, binding site, fatty acids, platelets

## Abstract

**Introduction:**

CD36 is a transmembrane glycoprotein involved in lipid uptake and signal transduction, playing a crucial role in various physiological and pathological processes. Structurally, it is composed primarily of an ectodomain (residues 30-439), which is essential for ligand binding and facilitating Long-Chain Fatty Acid (LCFA) uptake. Two lysine residues (K164 and K166) have been proposed to contribute significantly to LCFA internalization. Elucidating the ligand-binding cavities within CD36 can provide structural insights that may serve as a foundation for drug design.

**Methods:**

In this study, we employed a combination of molecular docking, molecular dynamics simulations, and cavity-detection algorithms to investigate the structural basis of CD36 interactions with small molecules and fatty acids.

**Results:**

We identified three main ligand-binding regions: a primary LCFA-binding cavity, a secondary LCFA-binding cavity, and a Small-Molecule Acceptor Cavity (SMAC). We evaluated three derivatives of the N-(2-hydroxy-4,6-dimethoxybenzylidene)acetohydrazide scaffold (AP5055, AP5156, and AP5258), two polyphenolic compounds (Puerarin and Salvianolic acid), and two well-known CD36 inhibitors (SSO and MTN).

**Discussion:**

Our findings implicate residues K334, E335, and R337 in disrupting LCFA uptake, while residues T195, L200, F201, Y202, P203, T207, A208, D209, Y230, and K231 delineate the initial segment of the SMAC.

**Conclusion:**

This study highlights critical residues and structural features involved in ligand recognition by CD36. The identification of the SMAC and its role in modulating LCFA uptake provides promising insights for the development of therapeutic agents targeting CD36-related pathologies, including cancer, diabetes, and metabolic disorders.

## 1 INTRODUCTION

CD36, a cell-surface receptor belonging to the scavenger receptor class B family, has attracted significant interest due to its crucial role in regulating physiological and pathological processes. Comprising 472 amino acid residues, CD36 is a 53 kDa transmembrane glycoprotein broadly expressed in multiple cell types, including monocytes, macrophages, subtypes of B and T lymphocytes, platelets, adipocytes, myocytes, epithelial, and vascular-endothelial cells [1-5]. Structurally, CD36 consists predominantly of an extracellular domain (ectodomain; G30-N439), anchored in the membrane by two transmembrane domains from N-ter, till the C-ter end (G8-V29 and L440-I461, respectively) [6]. Importantly, this receptor undergoes several Post- Translational Modifications (PTMs), including glycosylation, palmitoylation, ubiquitination, and phosphorylation, which directly influence its functions. Among these, N-linked glycosylation is essential for the receptor’s maturation, stabilization, trafficking, and surface expression [7]. Within the intracellular domain, two lysine residues (K469 and K452) have been described as critical polyubiquitination sites that target CD36 to proteasomal degradation [8]. Palmitoylation, another key PTM, occurs at four well-characterized sites (C3, C7, C464, and C466). These modifications influence an impaired receptor’s maturation, a deficient incorporation into lipid rafts, and, strikingly, the loss of the ability to induce cell surface localization of CD36 *via* insulin or AMPK-activation (AMP-activated protein kinase) [9-11]. CD36 binds to a wide array of extracellular ligands, which can be broadly categorized into three groups: 1) proteins containing Thrombospondin Structural Homology Repeat (TSR) domains, 2) Long-Chain Fatty Acids (LCFAs), and 3) molecules exhibiting molecular structures consistent with Danger-Associated or Pathogen-Associated Molecular Patterns (DAMPs and PAMPs) [12-15]. The ability of CD36 to bind LCFAs was first reported in 1993, and this function has since been recognized as central to fatty acid uptake in adipose and muscle tissues [16, 17]. Studies of insect orthologs revealed a role for CD36 in sexual behavioral responses to fatty-acid-based pheromones. In vertebrates, CD36-mediated signaling in taste buds and enteric cells in response to fatty acids contained in food was involved in food preference and neuroenteric signaling, further underscoring its dual role as both an LCFA transporter and signaling receptor [18-20]. Beyond its role in Fatty Acid (FA) transport, CD36 also functions as a signaling receptor that activates multiple pathways, including SRC family non-receptor tyrosine kinases (FYN and LYN), and serine/threonine kinases of the MAPK pathway (p38 and JNK) [21-23]. Both FA uptake/transport and FA-mediated signaling can be irreversibly blocked by long-chain FAs of *N*-hydroxysuccinimidyl esters (*e.g.*, sulfo-N-succinimidyl oleate, or SSO), which have been widely used to confirm the receptor’s role in these processes [24-27]. As a class B scavenger receptor member, CD36 exhibits a characteristic architecture: a large extracellular domain anchored to the membrane by two transmembrane helices and short cytosolic tails at both N- and C-termini [28, 29]. Furthermore, human CD36 is organized into five distinct regions: N-terminal cytoplasmic tail (residues 1-7), an N-terminal transmembrane domain (residues 8-29, also serving as a non-cleaved signal peptide), the ectodomain (residues 30-439), a C-terminal transmembrane domain (residues 440-461), and C-terminal cytoplasmic domain (residues 462-472). Among these, the ectodomain plays a vital role in ligand binding and facilitating fatty acid uptake [30]. Considerable efforts have been made to determine ligand-binding sites on CD36 at the atomic level. Two main binding regions have been proposed: the first accommodates Oxidized Low-Density Lipoprotein (oxLDL), Oxidized Phosphatidylcholine (oxPC), hexarelin, and FAs; the second interacts with thrombospondin and the malaria parasite *Plasmodium falciparum* [31-33]. A central cavity within the ectodomain has been identified, where two conserved lysine residues, K164 and K166, appear critical. One opening near K164 (entrance 1) is thought to serve as the primary entry point for membrane-distal fatty acids. A second opening (entrance 2), also located distally, allows fatty acids to transit through a tunnel-like pathway [34]. To further elucidate the role of K164 in LCFA internalization (specifically, Palmitic Acid (PLM)), Molecular Dynamics (MD) simulations have been employed. These studies revealed that initial recognition involves hydrophobic and electrostatic interactions between PLM’s hydrophobic tail and K164. The internalization pathway comprises a series of residues forming a hydrophobic portal, including R63, F65, L140, A143, A144, I148, L161, S162, K164, L187-L189, P191-T195, V198-F201, P203, Y204, T207, S268-I271, R273, I330, K334-S339, K385, L387, V389, L343, I341, T369, L391, L416, and E418 [35]. Additionally, the irreversible inhibition of CD36 by the membrane-impermeable SSO ester further emphasizes the essential role of K164. It is proposed that SSO covalently binds to K164 *via* an *N*-oleoyl amide linkage, disrupting both FA uptake and FA-induced Ca^2+^ signaling mediated by CD36 [36]. These findings collectively highlight K164 as a crucial residue in the fatty acid´s molecular recognition and internalization. However, identifying and characterizing additional binding sites for ligands with diverse chemical structures remains incomplete. Therefore, in this study, we employed molecular docking and Molecular Dynamics (MD) simulations, combined with cavity detection analyses, to characterize the binding modes of seven small molecules to CD36. We describe the interacting residues and chemical interactions that govern ligand recognition by the receptor.

## 2 METHODS

### 2.1 Binding Pocket and LCFA Cavity Detection

The CD36 structure (PDB entry 5LGD) was obtained from the RCSB PDB (https://www.rcsb.org/). After detailed visualization, all non-essential molecules unrelated to the receptor’s inherent activity (such as water molecules, cofactors, metals, and sugars) were removed. In particular, the two co-crystallized PLM molecules were removed to avoid interference with the analysis, as well as the CIDRa domain from MCvar1 PfEMP1. For this purpose, the protein structure was used without further processing. Detection of the probable LCFA acceptor cavities and small- molecule acceptor cavity in CD36 was carried out using two specialized web servers, CavityPlus (http://www.pkumdl.cn:8000/cavityplus/index.php#/) and POCASA (https://g6altair.sci.hokudai.ac.jp/g6/service/pocasa/). CavityPlus is a geometry-based method that uses a probe-sphere model to scan the protein surface, mimicking the kinetic behavior of small molecules as they bind to the protein. Hence, all the search parameters were kept at their default settings. Briefly, the algorithm first creates 3D grid points encompassing the entire protein, with a spacing of 0.5 Å in each grid point. A 10 Å radius probe sphere then identifies grid points it contacts, labeling them as “outside grid points,” while the remaining points are marked as “vacant grid points,” representing putative cavity spaces. Refinement of these cavities is performed using the “layer and depth” concepts combined with a “shrink-and-expand” algorithm [37]. The POCASA algorithm was applied according to our previous methodology [38].

### 2.2 Ligand Preparation

Ligand structures resulting from the rationale search of probable CD36 ligands (see Supplementary Material “Search for probable CD36 ligands” for details) were initially generated in 3D conformations using ChemSketch software (https://www.acdlabs.com/resources/free-chemistry-software-apps/chemsketch-freeware/). These structures were then converted to Z-matrix format using GaussView 5.0 [39] to delimit a geometrical and energetic minimization and correct proton assignment, using Gaussian 09 [40], under a semi-empirical AM1 method. The resulting output files were converted to .pdb format for subsequent docking calculations in Autodock (v4.2.6) [41]. For docking simulations with MOE [42], ligands were prepared using the same protocol but in .mol2 format.

### 2.3 Molecular Docking Protocols

Two methodological strategies were employed to validate and strengthen our analysis. Specifically, MOE and AutoDock (v4.2.6) programs were used to evaluate and characterize the potential binding interactions between the tested ligands and CD36. For both software programs, the docking simulations were designed to model two receptor states: CD36-unoccupied (receptor without bound PLM molecules) and CD36-occupied (receptor bound to two PLM molecules), based on the crystal structure of CD36 available in the RCSB-PDB (PDB entry: 5LGD). The MOE program was selected as the initial docking tool, considering the Triangle Matcher algorithm for initial ligand placement and the Rigid Receptor for refinement. The scoring functions used were the Affinity dG for initial pose generation (computing 30 poses), and GBVI/WSA dG for final refinement (computing 5 poses). As a complementary approach, the AutoDock (v4.2.6) program was employed, using parameters previously reported by our research group [38] (see Supplementary Material “Molecular Docking protocol with ADT software” for details) with a minor modification: the grid box was set at 126 Å^3^ across the X, Y, and Z dimensions (blind docking procedure), thereby encompassing nearly the entire ectodomain of the protein. Ligand-receptor interactions were visualized and analyzed using the Ligand interactions tool in MOE (for 2D representations, data not shown) and Pymol v0.99 [43] (for 3D structural interpretation).

### 2.4 Molecular Dynamics Simulation with MMGBSA Approach

Receptor-ligand complexes were prepared using *antechamber* and *tleap* modules of the AMBER22 software package (Case *et al.*, 2005). Systems were solvated using the TIP3P water model and neutralized with counterions by submersion in a truncated octahedral water box at 12 Å. The protein, water, and ions were defined by the ff14SB AMBER force field [44]. The General Amber force field (GAFF) was taken into consideration when defining the force field characteristics of ligands using AM1-BCC atomic charges [45]. Four thousand conjugate gradient cycles and five thousand steepest descent cycles made up the minimization. In an NVT ensemble with a Berendsen thermostat, the temperature was raised from 0 to 310K over 200 ps [46]. A Langevin thermostat [47, 48] and a Berendsen barostat [46] were used to equilibrate the density for 200 ps under an NTP ensemble. Using a Langevin thermostat and a Berendsen barostat, 100 ns long molecular dynamics (MD) simulations with a time step of 2 fs were run at 310 K under an NPT ensemble. The Particle Mesh Ewald approach was used to define the electrostatic term, and a 10 Å cutoff was used for non-bonded interactions [49]. The SHAKE algorithm [50] was used to constrain bond lengths at specific equilibrium values. The cpptraj function in the Amber 22 software was used to analyze Root-Mean-Square Deviation (RMSD), radius of gyration (Rg), Root-Mean-Square Fluctuations (RMSF), and clustering. To evaluate the binding free energy (ΔGbind), the Molecular Mechanics Generalized Born Surface Area (MMGBSA) method [51] was used. Using implicit solvent models [52], a 0.10 M salt concentration, and a total of 2000 snapshots, this analysis was conducted over the final 20 ns of the equilibrated simulation. As previously mentioned, the ΔGbind values were calculated [53].

## 3 RESULTS

### 3.1 Cavity-Finding Algorithms Identify A Primary and Secondary LCFA Entrance and A Small-Molecule Acceptor Cavity

Analysis of the potential cavities found in CD36 using the CavityPlus and POCASA algorithms revealed a converging consensus on one main primary and one secondary LCFA entrance (Figs. **1A**-**F**), as well as an internal cavity likely involved in small-molecule binding (Fig. **1A**). The primary LCFA entrance is in the outer region of the ectodomain of CD36, and its position aligns with the binding site of Phospholipid Molecule (PLM) observed in the co-crystallized CD36 structure from the RCSB-PDB database (PDB ID: 5LGD), where PLMs were only retained to aid visualization. This main entrance is demarcated by the result yielded for CavityPlus (ranked 7) and is characterized according to the arrangement of the residues L140, A141, V142, A144, A145, I148, L187, P193, V194, T195, T196, T197, V198, E335, G336, and R337 (Fig. **1C**). Moreover, the pocket deep center (ranked 2) by POCASA algorithm, reinforces the characterization of this entrance highlighting the residues I148, V194, T195, R337 and the additional aromatic residue Y149, which collectively contributes to the initial molecular recognition of LCFA molecules such as PLM. Noteworthy, the presence of two charged residues, E335 and R337, which appear to form a stabilizing salt bridge delimiting the beginning of a funnel-shaped cavity (Fig. **1B**). This funnel extends from the LCFA binding entrance toward the membrane´s outer leaflet, terminating near the two alpha helices that anchor the receptor to the membrane (Fig. **1B**). One of these helixes includes residues N428, M429, F430, R431, S432, Q433, and V434 (C-terminal region), while the other comprises residues T48, I49, A50, F51, K52, N53, W54, V55, and K56. The aforementioned correlates structurally with the bottom of the largest cavity detected by CavityPlus (result number 3), which may define the path for LCFA internalization across the protein structure and into the membrane. According to POCASA, the two highest-ranking deep centers are at the beginning of the primary LCFA entrance (ranked 2) and the internal funnel described above (ranked 1). These findings correlate with the CavityPlus results, ranked 3 (associated with the secondary LCFA entrance) and 4 (spanning nearly the entire protein), which encompass many CD36 residues. Interestingly, the intersection of cavities 3 and 4 in the CavityPlus results defines a prominent central binding site. We designated it as the Small-Molecule Acceptor Cavity (SMAC). The entrance to SMAC is formed by residues T195, Y202, P203, T207, A208, K231, and E335 (Fig. **1D**). Additionally, a secondary LCFA entrance was identified in the lateral middle region of the receptor, as part of CavityPlus result ranked 3 (Fig. **1E**). This secondary site is delineated by residues A299, F300, F312, G326, V327, and L328, suggesting an alternative molecular recognition route for LCFA based on receptor orientation at the plasma membrane (Fig. **1F**). This site is further supported by POCASA´s pocket deep center ranked 3 (coordinates: -46.4 -15.3 32.7) reinforcing the premise that CD36 can accommodate two LCFA molecules simultaneously, guiding them through the inner funnel that runs from the top region or the lateral side of CD36 towards the two alpha helix regions of the end terminals of the receptor, as observed in the crystal structure (PDB ID: 5LGD).

### 3.2 SMAC Delimits LCFA Internalization in the Unoccupied Conformation of CD36

Docking analysis using MOE software of the two polyphenolic compounds (Puerarin and Salvianolic acid) and the CD36 inhibitor SSO revealed a well-defined cavity near to the central region of CD36 in the unoccupied conformation (Fig. **2A**). In that way, the glycosidic moiety of Puerarin interacts with E335 through two h-bonds (2.84 and 2.94 Å), underscoring the importance of these residues in the initial LCFA uptake process. Interestingly, the ring C of the flavan core of Puerarin appears to influence LCFA internalization primarily through steric hindrance. It establishes h-bonds with the NH_2_ group of R63 (3.52 - 3.82 Å) and engages in electrostatic interactions with the negatively charged residues D209 and D270, in addition to π-interactions involving the benzene ring of the C-ring (Fig. **2B**). Besides, SSO primarily interacts with residues that hinder the arrangement of LCFAs required for their progression from the entrance of the primary LCFA cavity to the receptor’s midsection during the initial steps of internalization. This is evident from the binding mode of SSO, in which its backbone forms aliphatic interactions with L200 and F201. Additionally, the 2,5-dioxopyrrolidine-3-sulfonate moiety of SSO orients towards critical residues that constitute the entrance of the SMAC including D209 and Y230, which altogether with T195, Y202, P203, T207, A208, K231, and E335 define the initial segment of this cavity (Fig. **2C**). Moreover, SMAC is further characterized by the orientation of one of Salvianolic´s acid dihydroxy phenyl rings toward key residues K334, E335 and R337. These residues form a salt-bridge that plays a key role in molecular recognition of LCFA by reorienting early internalization steps *via* electrostatic interactions between the phenolic hydroxyl groups and the NH2 group of R337, potentially disrupting the salt bridge with E335 (Fig. **2D**). Lastly, MTN penetrates the inner region of the second LCFA acceptor cavity, interacting with residues that define the cavity´s structure. These include I341, S342, L343, and L387 in the central part, and D270, I271, I275, L295, T369, K385, R386, and L387 from the bottom part. Notably, I271 is a key stabilizing residue, forming two π-arene interactions with the aromatic portion of MTN (Fig. **2E**). All the non-bonded interactions from the MOE docking studies in the unoccupied conformation of CD36 are collected in Table **1**. From the ADT results (see Supplementary Material “Fig. **S1**” for details), it is worth mentioning that Puerarin, SSO, and Salvianolic acid exhibit a striking orientation at the SMAC region (see Supplementary Material “Fig. **S1A**” for details). Indeed, the binding mode of Puerarin is facilitated by polar interactions at the SMAC entrance. These include an H-bond between the glycosidic moiety and G199, and two additional H-bonds with Y202. Furthermore, an H-bond is formed between D270 and the hydroxyl group on the C-ring of Puerarin, highlighting the relevance of these residues in ligand anchoring (see Supplementary Material “Fig. **S1B**” for details). Moreover, the binding mode of SSO stands out due to its striking similarity to that of the PLM molecule (analyzed in the occupied conformation only for a better understanding of the molecular recognition mediated by CD36). However, SSO adopts a distinct orientation, with its aliphatic backbone directed toward the entrance of the LCFA acceptor cavity. Simultaneously, the 2,5-dioxopyrrolidine-3-sulfonate moiety extends toward key residues involved in the initial steps of the LCFA internalization, particularly K334. This interaction is stabilized through two metal/ion interactions and one h-bond with the side chain of K334, involving two oxygen atoms from the SO3- group and a carbonyl oxygen from the 2,5-dioxopyrrolidine-3-sulfonate moiety, respectively (see Supplementary Material “Fig. **S1C**” for details). Moreover, Salvianolic acid interacts with pivotal residues R337, K334, and E335, primarily through polar contacts involving carbonyl groups from both its carboxylic acid and ester moieties. These interactions once again underscore the significance of the salt bridge formed between E335 and R337 in the early steps of LCFA internalization mediated by CD36 (see Supplementary Material “Fig. **S1D**” for details). Finally, MTN, a well-known CD36 inhibitor, targets the receptor's lateral second LCFA-binding site. This secondary cavity is formed in its top region, comprising residues R273, A299, F300, N309, F312, G326, V327, and L328. Specifically, the 2-(methylthio)cyclohex-2-ene-1,4-dione moiety of MTN forms two h-bonds with the oxygen atoms of R273 and V327 at distances of 2.91 and 3.04 Å, respectively. Moreover, the methylthio substituent is oriented toward the inner region of the second LCFA entrance, suggesting a mechanism that may hinder the internalization of LCFA-like molecules such as PLM, but as a secondary mechanism occurring after the primary LCFA cavity has engaged (see Supplementary Material “Fig. **S1E**” for details). All non-bonded interactions from the ADT docking simulations in the unoccupied conformation of CD36 are presented in Table **S1** in the Supplementary Material. Given the relevance of the MOE-derived results and the clear definition of the small-molecule acceptor cavity by the ligands Puerarin, Salvianolic, and SSO, only these binding topologies were selected for further MD simulations.

### 3.3 Derivatives of N-(2-hydroxy-4,6-dimethoxybenzylidene)acetohydrazide Scaffold Target the Inner Site of the SMAC in the Unoccupied Conformation of CD36

Analysis of the binding modes of the three N'-(2-hydroxy-4,6-dimethoxybenzylidene)acetohydrazide derivatives (AP5055, AP5156, and AP5258) using MOE software reveals a well-defined cavity closely associated with the main entrance of PLM in the unoccupied conformation of CD36 (Fig. **3A**). Indeed, the SMAC appears to be constituted by residues involved in the overlapping binding poses of these ligands. In detail, AP5055 and AP5156 share a highly similar binding mode, involving interactions with key residues such as N118, R63, F201, P203, D270, C333, K334, R337, L416, and E418 (Figs. **3B**, **C**). Notably, the benzo[*b*]thiophene and quinoline moieties from AP5055 and AP5156, respectively, are oriented toward the PLM aliphatic tail (based on comparison between the unoccupied and occupied conformations of the receptor and only for a better understanding of the molecular interactions), potentially obstructing the passage of PLM to the middle part of the funnel. This blockage could impair the internalization of the FA. Similarly, the imidazo [1,2-a]pyridine moiety of AP5258 is positioned to form a double π-arene interaction with F201, a key interaction that may inhibit fatty acid translocation from the primary LCFA entrance to the inner region of CD36. Importantly, the 2-hydroxy-4,6-dimethoxybenzylidene moiety of AP5258 extends into the lower portion of the primary LCFA entrance, interacting with aliphatic residues L140, A141, A144, V198, L391, and V339, as well as the polar uncharged residue T197 and the positively charged R337 (Fig. **3D**). These residues are likely involved in the initial stages of LCFA internalization. Furthermore, the side chain of R337 adopts an orientation that may enable a π-cation interaction with the aromatic ring of the 2-hydroxy-4,6-dimethoxybenzylidene moiety in AP5258, contributing to enhanced binding energy relative to AP5055 and AP5156. Besides, the benzo[*b*]thiophene and quinoline moieties from AP5055 and AP5156 are stabilized by a cluster of charged residues. Specifically, interactions are observed with positively charged residues R63, R96 and K385, likely through π-cation interactions, and with negatively charged residues D270 (π-arene interaction in AP5055) and E418 (Fig. **3B**). In the case of AP5156, a similar interaction is formed with Y204 instead of D270 (Fig. **3C**). Remarkably, these specific interactions are absent in AP5258, likely due to the lack of an aromatic system equivalent to those in AP5055 and AP5156. The imidazo [1,2-a]pyridine moiety in AP5258 appears unable to engage in similar electrostatic interactions, possibly explaining differences in binding profiles and affinity (Fig. **3D**). All the non-bonded interactions from the MOE docking studies in the unoccupied conformation of CD36 are collected in Table **1**. According to the ADT results, the binding modes of AP5055, AP5156, and AP5258 are located slightly upstream and oriented toward the entrance of the SMAC region (see Supplementary Material “Fig. **S2A**” for details), compared to the results obtained with MOE. Notably, these binding modes establish interactions with key residues at the SMAC entrance, including T195, Y202, P203, and T207. Specifically, binding mode analysis of AP5055 and AP5156 reveals that their benzo[b]thiophene and quinoline moieties, respectively, are spatially positioned near the carboxylic acid of the PLM molecule (when compared with PLM bound in the occupied conformation of CD36 used solely for visualization and comparative purposes). AP5055 and AP5156 are primarily stabilized through h-bonds with residue G199 and probable π-cation interactions with the positively charged residue R337, which interacts with their aromatic moieties. These interactions emphasize the importance of R337 in the molecular recognition of LCFAs and suggest that binding of these ligands could interfere with the receptor’s ability to internalize such molecules (see Supplementary Material “Figs. **S2B**, **C**” for details). Interestingly, AP5258 adopts a slightly lower binding position compared to its corresponding MOE docking output but still defines a core interaction pattern shared among all N-(2-hydroxy-4,6-dimethoxybenzylidene)acetohydrazide scaffold derivatives. This interaction involves the 2-hydroxy-4,6-dimethoxybenzylidene moiety of all three ligands and residues G199, L200, Y202, K334, R337, and V339, highlighting a conserved orientation that may be critical for ligand recognition and stabilization within the SMAC region (see Supplementary Material “Fig. **S2D**” for details). All non-bonded interactions from the ADT docking studies in the unoccupied conformation of CD36 are presented in Table **S1** in the Supplementary Material section 3. Due to the robustness of the MOE-derived binding modes and the ability of the N-(2-hydroxy-4,6-dimethoxybenzylidene)acetohydrazide scaffold derivatives to effectively describe the inner architecture of the SMAC, only these docking output topologies were selected for further MD simulations.

### 3.4 SMAC Delimits the Main Binding Site in the Occupied Conformation of CD36

Binding modes analysis of the tested ligands using MOE software, in the occupied conformation of CD36, highlights once again the pivotal role of the SMAC region in molecular recognition towards ligands structurally distinct from LCFAs (Fig. **4A**). In essence, four of the tested ligands target a binding site located near the tail of the PLM molecule, situated in the upper region of the LCFA receptor entrance. This binding orientation is delimited by the overlap of the benzo[b]thiophene, quinoline and C-ring moieties of AP5055 (Fig. **4B**), AP5156 (Fig. **4C**) and Puerarin (Fig. **4D**), respectively, as well as by the slightly more distal aliphatic tail of SSO (Fig. **4E**). The primary residues involved in stabilizing these moieties include F201, P203, D209, D270 (for AP5055, *via* a π-arene interaction) (Fig. **4B**); L416, Y204 and E418 (the latter two interacting with AP5156 through a π-arene and a h-bond, respectively) (Fig. **4C**). Notably, the glycosidic moiety of Puerarin forms nearly identical interactions as observed in the unoccupied conformation of CD36. In addition, engaging with the residue cluster C333, K334, and E335 (most prominently with E335, which forms two h-bonds (2.85 - 2.94 Å). Additionally, the C-ring of Puerarin is oriented towards R63, stabilized by two h-bonds (3.52 - 3.81 Å) (Fig. **4D**). Regarding SSO, its aliphatic tail is oriented to the backbone of the PLM molecule by establishing aliphatic interactions, also interacting with a cluster of residues including L200, F201, Y202, and P203 mainly by hydrophobic interactions. Lastly, is important to note that the 2,5-dioxopyrrolidine-3-sulfonate moiety of SSO is oriented to the SMAC entrance interacting with essential residues for initial molecular recognition as G210, V211, Y230, K231, and G232 (Fig. **4E**). Moreover, the Salvianolic acid adopts a binding topology where one of its di-hydroxylated benzene rings is oriented toward the critical residues K334, E335 and R337. Notably, R337 forms two h-bonds (3.06 - 3.13 Å), which may interfere with the salt-bridge established between E335 and R337, potentially disrupting early LCFA internalization steps mediated by CD36 (Fig. **4F**). Interestingly, AP5258 binds in a distinct location not observed for any of the other ligands in either the occupied or unoccupied conformations of CD36. In summary, its binding mode is positioned near the primary LCFA-binding cavity, establishing contacts with residues L187, Y192, P193, V194, T195 (*via* π-arene interactions), T196, and T197 (Fig. **4G**). It is striking that, among all the tested ligands, Y202 is the only residue consistently involved in nearly all binding modes. The above interaction is likely driven by the complementarity between the chemical structure of AP5258 and the surrounding binding site residues. This complementarity influences its binding energy and is reflected in its lesser exergonic (-5.32 kcal/mol) compared to the other derivatives of N-(2-hydroxy-4,6-dimethoxybenzylidene)acetohydrazide scaffold (AP5055 (-5.73 kcal/mol) and AP5156 (-5.76 kcal/mol)). Ultimately, MTN reaches the inner region of the secondary LCFA acceptor cavity, where it is stabilized by aromatic and aliphatic residues, including F266, F300, I271, I275, L295, L328, I341, L343, and L387. Additionally, polar uncharged and charged residues (S268, S342, D270, K385, and R386) contribute to ligand stabilization (Fig. **4H**). Altogether, these interactions yield a favorable binding energy of -5.39 kcal/mol, indicating the cavity’s capacity to bind molecules primarily through aliphatic interactions and, secondarily, through polar interactions. All the non-bonded interactions from the MOE docking studies in the occupied conformation of CD36 are collected in Table **2**. Concerning the ADT docking results for the occupied conformation of CD36, only SSO and AP5258 reached the inner region of the SMAC in a manner highly consistent with previously reported binding modes for the same ligands in the unoccupied conformation. In contrast, the remaining ligands bound to a different site, as opposed to the one identified using both MOE and ADT algorithms (see Supplementary Material “Figs. **S3A**-**H**” for details). Specifically, AP5055 oriented its benzo[b]thiophene moiety in a manner similar to Puerarin, with its glycosidic moiety directed toward residues C313 and T314. In particular, T314 engaged through π-arene interactions with both AP5055 and Puerarin (see Supplementary Material “Figs. **S3B** and **D**” for details). Additionally, alternative interactions mainly involve negatively charged residues such as E240 (via h-bond with AP5055) (see Supplementary Material “Fig. **S3B**” for details), D329 (via h-bond with Puerarin and three polar contacts with Salvianolic) (see Supplementary Material “Figs. **S3D** and **F**” for details). Although these interactions may contribute to the ligand´s binding energy, their location outside the SMAC or the primary LCFA entrance suggests a less reliable or physiologically relevant binding mode, especially considering that most of the previously analyzed binding modes consistently converged within the SMAC region, delimiting it as the principal site for small-molecule interactions with CD36. In contrast, AP5156 demonstrates binding at the secondary LCFA subcavity, also identified in CavityPlus result 3. This subcavity, located on the lateral side of CD36, has been implicated in the internalization of a second LCFA molecule, as inferred from the nearby position of the PLM molecule in the receptor´s crystal structure. Entrance to this subcavity is defined by residues A299, F300, F312, G326, V327, and L328, which interact directly with the quinoline moiety of AP5156 (see Supplementary Material “Fig. **S3C**” for details). Additionally, aliphatic interactions occur between I271 and L343 and the aromatic ring of the same moiety at the bottom of the subcavity. Notably, the carboxylic acid group of PLM is located within 3 Å of AP5156, strongly suggesting that this subcavity may serve as an alternative entrance for LCFA, an entry point that could be blocked by ligands capable of occupying the subcavity’s entrance. Besides, SSO adopts a conformation that spans from the SMAC to the PLM molecule, located at the primary LCFA entrance, through aliphatic interactions. This ligand establishes interactions with several key residues, including K334, which fully engages with the sulfonate moiety of SSO *via* three h-bonds (2.61, 2.83, and 3.42 Å), E335 and R337, potentially disrupting the salt-bridge formed between these residues and thereby interfering with the early steps of LCFA internalization (see Supplementary Material “Fig. **S3E**” for details). Additional interactions occur with Y202 and D270, with a h-bond at 3.29 Å. Similarly, AP5258 adopts a binding mode located slightly deeper within the SMAC, though establishing fewer interactions with the aforementioned key residues, interacting primarily with K334, D270, and Y202. Notably, the imidazo [1,2-a]pyridine moiety of AP5258 is oriented to establish a π-arene with S269 and π-cation with R96, highlighting these residues as characteristic of the mid-region of CD36 (see Supplementary Material “Fig. **S3G**” for details). Finally, the binding mode of MTN displays a conformation highly similar to that observed in the unoccupied conformation of CD36. Specifically, R273 and V327 form h-bonds (3.27 and 2.91 Å, respectively) with the two oxygen atoms of the 2-(methylthio)cyclohex-2-ene-1,4-dione moiety of MTN (see Supplementary Material “Fig. **S3H**” for details). Additionally, the methylthio substituent is oriented toward the inner part of the subcavity. Based on the docking results for MTN in both conformations, this ligand emerges as a promising inhibitor targeting the secondary LCFA subcavity. All non-bonded interactions from the ADT docking studies in the occupied conformation of CD36 are presented in Table **S2** in the Supplementary Material. Given the relevance of the MOE-derived results in defining the SMAC as the primary small-molecule binding site on CD36, only the topologies output by MOE were selected for subsequent Molecular Dynamics (MD) simulations.

### 3.5 Stability of the Simulated Systems

The mobility of the receptor-ligand complexes was evaluated through RMSD and Rg analyses (Figs. **5A**-**C**, **6A**-**C**). Notably, in the unoccupied conformation of the receptor-ligand complexes, a stable behavior was observed between 50 and 80 ns, as indicated by the RMSD (Fig. **5A**) and Rg (Fig. **5B**) profiles, therefore, the last 20 ns of each trajectory were selected to carry out these analysis, thus, the mean ± SD of each receptor-ligand complex is collected in Table **3**. Rg values during this interval ranged from 22.6 to 22.8 Å. Among the tested ligands in the unoccupied conformation, AP5258 exhibited the highest degree of fluctuation, possibly due to its initial topology, which adopts a binding mode extending from the main primary LCFA entrance and the inner part of the SMAC. This orientation leads to a re-accommodation that shifts the ligand toward the SMAC entrance, highlighting its relevance to the molecular recognition of CD36 by non-LCFA ligands, while simultaneously impairing its ability to attain stability within the ligand-receptor complex. However, it showed a tendency to compact its structure during specific intervals (40 - 60 ns and 60 - 80 ns), probably due to its ability to reach and interact with residues located at the lower section of the primary LCFA entrance, including L140, A141, A144, V198, L391, and V339. Additionally, polar interactions formed with the polar uncharged residue T197 and the positively charged residue R337. This compaction tendency appears to be reinforced by a π-cation interaction between the aromatic system of the 2-hydroxy-4,6-dimethoxybenzylidene moiety of AP5258 and R337. This interaction may contribute to the improved binding energy of AP5258 (-7.28 kcal/mol), as determined by MOE docking, compared to other N-(2-hydroxy-4,6-dimethoxybenzylidene)acetohydrazide scaffold derivatives, AP5055 (-5.73 kcal/mol) and AP5156 (-5.76 kcal/mol). For the remaining ligands (Puerarin, Salvianolic, and SSO), no significant fluctuations were observed in any of the analyzed parameters, indicating stable binding within the well-defined SMAC region, consistently maintained interactions with essential stabilizing residues, including L140, A144, L200, F201, Y202, K334, E335, R337, V339, and L391. Such consistent interactions support their classification as cognate ligands of CD36. In the occupied conformation of CD36, receptor-ligand complexes showed Rg values ranging from 22.7 to 23.0 Å, with both RMSD (Fig. **6A**) and Rg (Fig. **6B**) profiles reaching stable behavior between 50 and 80 ns. As a result, only the final 20 ns of this equilibrated timeframe were used for further analyses; thus, the mean ± SD for each receptor-ligand complex is reported in Table **3**. A notable exception was observed in the RMSD trace for the AP5055-CD36 complex, which exhibited a high degree of mobility. Strikingly, this behavior may be attributed to a conformational rearrangement that occurs after the ligand binds to the SMAC-interacting residues, thereby increasing the flexibility of the complex. In contrast, the remaining ligands that shared almost the same binding pose (AP5156, Puerarin, SSO, and Salvianolic acid) exhibited relatively stable RMSD traces from approximately 50 ns onward, maintaining this behavior throughout the simulation. The RMSF analysis further supported these findings. In detail, a pronounced fluctuation peak was observed for residues S269 and D270 in the AP5055-CD36 complex. These residues interact with the benzo[*b*]thiophene of AP5055, likely leading to steric clashes and contributing to the observed fluctuation peak, an expected result given the unfavorable nature of interactions between polar residues and the aromatic moiety of AP5055. Consequently, RMSF analysis was performed for both occupied (Fig. **5C**) and unoccupied (Fig. **6C**) receptor conformations over the last 20 ns of the equilibrated simulation.

### 3.6 Binding Free Energy of Protein-Ligand Complexes

The MMGBSA approach was employed to evaluate the thermodynamic components of ligand-receptor binding. Throughout the simulation period, all tested compounds exhibited negative binding free energy, indicating favorable interactions with CD36 (Table **4**). The non-polar contributions (ΔEvdw + ΔGnpol, sol) emerged as the primary stabilizing forces for ligand binding within the receptor. Overall, all compounds demonstrated a more favorable binding affinity in the unoccupied conformation of CD36 compared to the occupied conformation. Among them, AP5055, AP5156, and AP5258 showed the most significant improvement in binding energy in the unoccupied conformation. Conversely, in the occupied conformation, Puerarin, AP5156, and AP5258 displayed the strongest binding affinities. Notably, the binding mode analysis in the unoccupied conformation revealed a conserved cluster of residues across all ligand-receptor complexes. These residues were also observed in the initial topologies obtained from MOE docking simulations, underscoring their pivotal role in molecular recognition of CD36 ligands and in maintaining ligand engagement within the SMAC. In particular, AP5055 and AP5156 reorient their benzo[*b*]thiophene and quinoline moieties, respectively, toward the main LCFA entrance (Figs. **7A**, **B**). This behavior may occur as a mechanism when the early internalization steps for LCFA are interrupted. Similarly, AP5258 reoriented its imidazo [1,2-a]pyridine moiety toward the SMAC entrance (Fig. **7C**). All the three ligands maintained significant interactions with pivotal residues such as L140, V198, and R337, while AP5055 and AP5156 also contacted residues A141, V142, A144, A145, I148, L187, and V194, suggesting that these residues may play a critical role in modulating LCFA internalization. A particularly important observation across all ligands was the consistent interaction with K334, E335, and R337, residues that may contribute to the disruption of LCFA uptake, potentially by interfering with the salt bridge formed between E335 and R337 (Figs. **7A**-**F**). Besides, in the occupied conformation of CD36, an overlapping set of conserved residues was consistently observed across all complexes. Specifically, N118, F201, T207, A208, and D209 were in close contact with all the ligands, marking them as essential binding determinants in this conformational state (Figs. **8A**-**F**). Moreover, the residues K334, E335, and R337, previously highlighted in the unoccupied conformation, remained conserved in the occupied conformation for all ligands except AP5055, reinforcing their critical role in ligand recognition and stabilization within the SMAC. Lastly, it is worth noting that in the occupied conformation, the complexes generally exhibited less favorable binding energies during MD simulations. This may be attributed to the presence of PLM molecules, which appear to induce conformational changes in CD36 that prevent optimal ligand accommodation within SMAC. This effect was evident in the binding modes observed during simulation, where ligands were often displaced from the inner SMAC region, unlike in the unoccupied conformation, where deeper binding was consistently achieved. All non-bonded interactions identified in MD studies are listed in Table **5**.

## 4 DISCUSSION

Previous studies have described the pivotal role of K164 and K166 in the molecular recognition of LCFAs, such as Palmitic Acid (PLM), by CD36, supported by functional assays including Mass Spectroscopy, ELISA, and others [36, 54]. While substantial information is available regarding the specific involvement of K164, there remains a notable gap in the description of the residues forming the cavities responsible for LCFAs' internalization, as well as the molecular nature of the interactions governing the early stages of recognition and processing within the receptor. Therefore, our results, obtained through the identification of potential cavities within the CD36 structure, combined with docking (employing two search algorithms) and molecular dynamics (MD) simulations, provide compelling evidence for the existence of two LCFAs acceptor cavities and a distinctive Small-Molecule Acceptor Cavity (SMAC). Specifically, the primary LCFA acceptor cavity is located slightly distant from K164 and K166, and is encompassed by residues L140, A141, V142, A144, A145, I148, L187, P193, V194, T195, T196, T197, V198, E335, G336, and R337. Notably, these residues have not been previously reported as critical participants in LCFA molecular recognition. In addition, a secondary LCFA acceptor cavity was identified, corresponding to a region previously suggested to accommodate a second PLM molecule [55] and formed by residues A299, F300, F312, G326, V327, and L328, potentially facilitating the internalization of a second LCFA molecule, a finding supported by analysis of the crystal structure of CD36 [34]. Moreover, cavity-finding algorithms also identified a well-defined Small-Molecule Acceptor Cavity (SMAC). This cavity is supported by the binding modes observed for the tested ligands in both receptor conformations (unoccupied and occupied). Its entrance is delimited by residues T195, Y202, P203, T207, A208, K231, and E335. While many of these residues have been previously linked to various functional roles of CD36, there have been no reports explicitly describing their collective or individual roles in forming a LCFA-binding cavity or a small- molecule binding site for ligands distinct from LCFAs [35]. Interestingly, the proposed mechanism of inhibition for well-characterized CD36 inhibitors, such as SSO and MTN, does not align with the insights from our analyses. In that sense, it has been suggested that SSO inhibits CD36 *via* covalent modification of K164, forming an *N*-oleoyl amide through a reaction between its sulfo-N-succinimidyl oleate ester and this residue. However, our results did not predict any plausible interaction between SSO and K164 (or K166, another residue previously proposed to be critical for LCFA molecular recognition36). Instead, SSO was found to bind in the SMAC, occupying the unoccupied conformation of CD36 and orienting its 2,5-dioxopyrrolidine-3-sulfonate moiety toward key residues such as D209 and Y230. Meanwhile, in the occupied conformation, it was possible to observe aliphatic complementarity with the SSO backbone, and the PLM tail observed in the crystal structure of CD36 [34]. This suggests that SSO could be stabilized in the occupied conformation by interacting with both the receptor and the co-bound LCFA. Moreover, in the case of MTN, although it is a well-established functional inhibitor of CD36, little is known about the specific residues involved in its molecular recognition. Our results revealed that MTN targets the secondary LCFA acceptor cavity located on the lateral side of CD36. The upper region of this cavity comprises residues A299, F300, F312, G326, V327, and L328, while the bottom involves residues I271 and L343. These findings reinforce our previous observations on the probable binding mode of MTN in CD36 [56]. Overall, our results indicate that K164 and K166 may not play a pivotal role in the molecular recognition of LCFAs by CD36, and these residues are not the molecular targets of well-known CD36 inhibitors such as SSO and MTN. In this regard, although some experimental assays have highlighted the pivotal role of these lysine residues in the molecular recognition of CD36 toward its cognate ligands, such as SSO and MTN, the evidence remains inconclusive. Our results instead point to alternative residues that form more suitable binding cavities, which do not involve K164 and K166 as key residues, thereby delimiting the primary binding sites for new drug design or drug repurposing protocols. In that sense, our analysis highlights the identification and characterization of the SMAC, whose ability to bind ligands of non-LCFA chemical nature positions it as an attractive pharmacological site for novel drug discovery and the rational design of pharmacophores.

## CONCLUSION

The ongoing search for novel pharmacological targets to treat a wide range of diseases, including cancer, diabetes, and autoimmune disorders, continues to evolve rapidly. In this context, CD36 has emerged as a promising molecular target due to its multifaceted roles, including facilitating Fatty Acid (FA) transport and participating in the transduction of several key signaling pathways. These include the activation of SRC family non-receptor tyrosine kinases (FYN and LYN), and serine/threonine kinases of the MAPK pathway (p38 and JNK), all of which are essential for maintaining cellular homeostasis and whose dysregulation leads to disease states. In the present study, we employed a comprehensive computational approach combining molecular docking, molecular dynamics simulations, and protein cavity detection to describe, at the atomistic level, two LCFA acceptor cavities and a Small-Molecule Acceptor Cavity (SMAC) within CD36, which can bind ligands of different chemical natures than LCFA. Notably, our results highlight the critical role of a salt bridge between residues E335 and R337 in disrupting the initial steps of LCFA internalization once this ligand enters the primary LCFA acceptor cavity. This cavity is mainly formed by residues L140, A141, V142, A144, A145, I148, L187, P193, V194, T195, T196, T197, V198, E335, G336, and R337. Adjacent to this region, the SMAC is defined by several essential residues as R63, N118, T195, Y202, P203, T207, A208, D209, Y230, K231, D270, K334, E335, R337, L416, and E418, which may play a regulatory role in CD36-mediated LCFA internalization. Furthermore, we identified a secondary LCFA acceptor cavity accessible *via* the CD36 inhibitor MTN. This cavity is composed of residues D270, I271, R273, I275, L295, A299, F300, N309, F312, G326, V327, L328, I341, S342, L343, T369, K385, R386, and L387. Altogether, these findings offer new insights into the key structural features and binding determinants of CD36 that could be exploited in the rational design of pharmacophores as well as for the development of novel therapeutic agents, either through de novo drug design or drug repurposing targeting CD36, with potential applications in treating a variety of diseases where CD36 dysfunction has been implicated.

## STUDY LIMITATIONS

This study is entirely computational and thus constrained by the inherent limitations of in silico methodologies. The atomistic characterization of the CD36 binding site is derived from cavity detection, molecular docking, and molecular dynamics simulations, all of which depend on the accuracy of the structural model, parameterization, force fields, and scoring functions employed. Predicted binding poses and calculated interaction energies represent theoretical approximations and may not directly translate into experimental affinities or functional modulation. In the absence of biochemical or cellular validation, these findings should be considered hypothesis-generating and require experimental confirmation.

## Figures and Tables

**Fig. (1):**
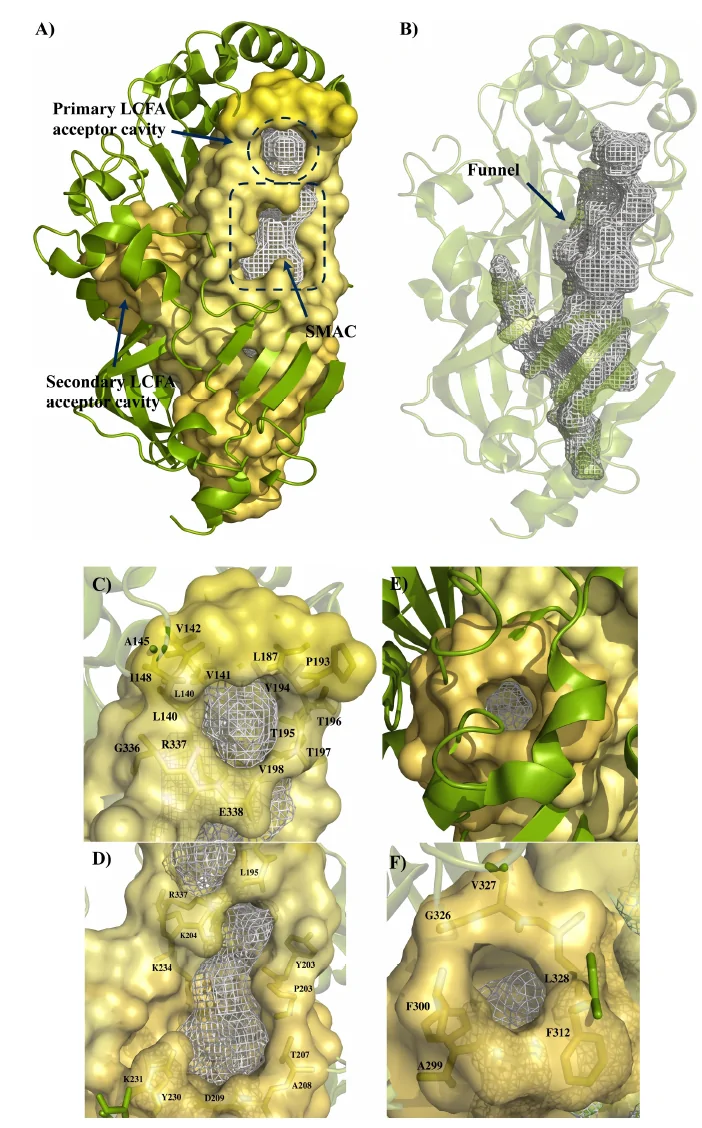
CD36 cavities. (**A**) Main cavities on CD36, (**B**) Funnel connecting the primary LCFA with the bottom of the receptor, (**C**) Residues forming the Primary LCFA entrance, (**D**) Residues forming the Small-Molecule Acceptor Cavity, (**E**) Secondary LCFA entrance in a general perspective (the image in (A) Was rotated 45° for a better view), and (**F**) Residues forming the Secondary LCFA entrance.

**Fig. (2):**
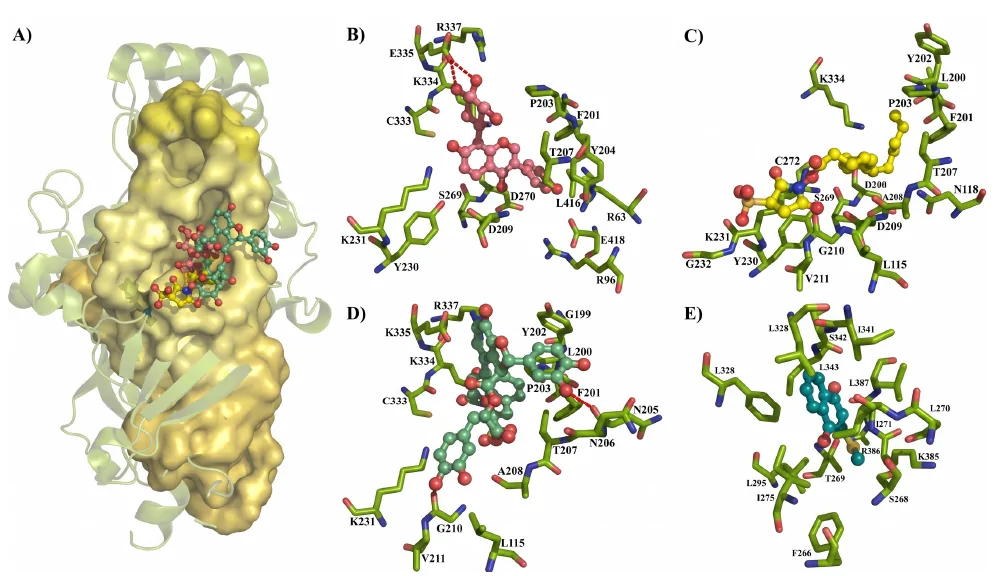
Puerarin, SSO, Salvianolic acid, and MTN docked on the unoccupied conformation of CD36 yielded by MOE software. (**A**) Overlapping of the binding modes from the assayed ligands depicting mostly the interaction with the SMAC, (**B**) Binding mode of Puerarin, (**C**) Binding mode of SSO, (**D**) Binding mode of Salvianolic acid, and (**E**) Binding mode of MTN. H-bonds are depicted as red dashes.

**Fig. (3):**
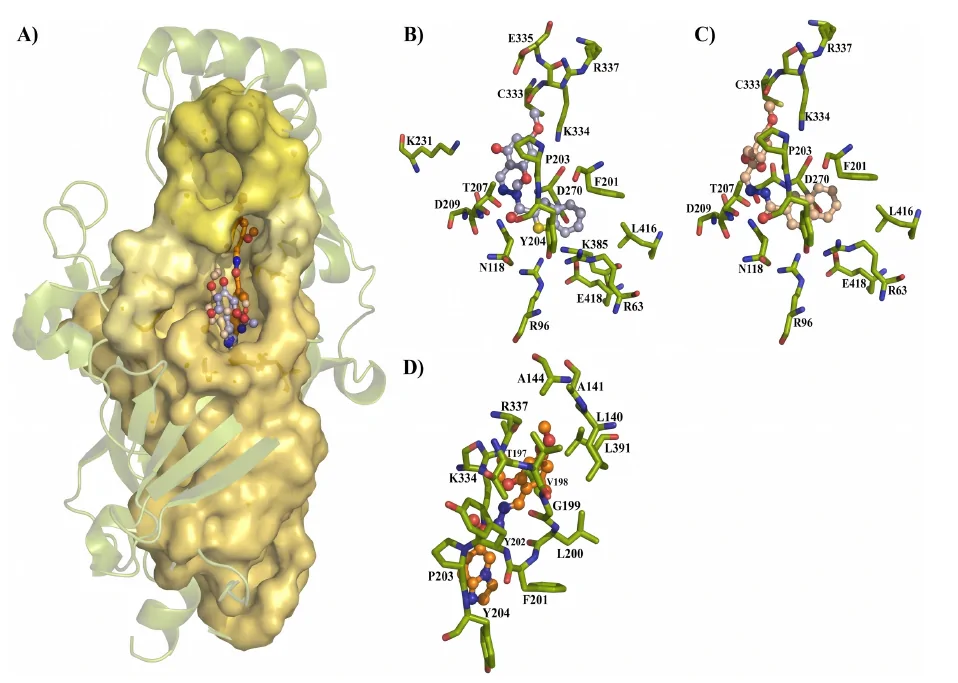
AP5055, AP5156, and AP5258 docked on the unoccupied conformation of CD36 yielded by MOE software. (**A**) Overlapping of the binding modes from the assayed ligands depicting the interaction with the SMAC, (**B**) Binding mode of AP5055, (**C**) Binding mode of AP5156, and (**D**) Binding mode of AP5258.

**Fig. (4):**
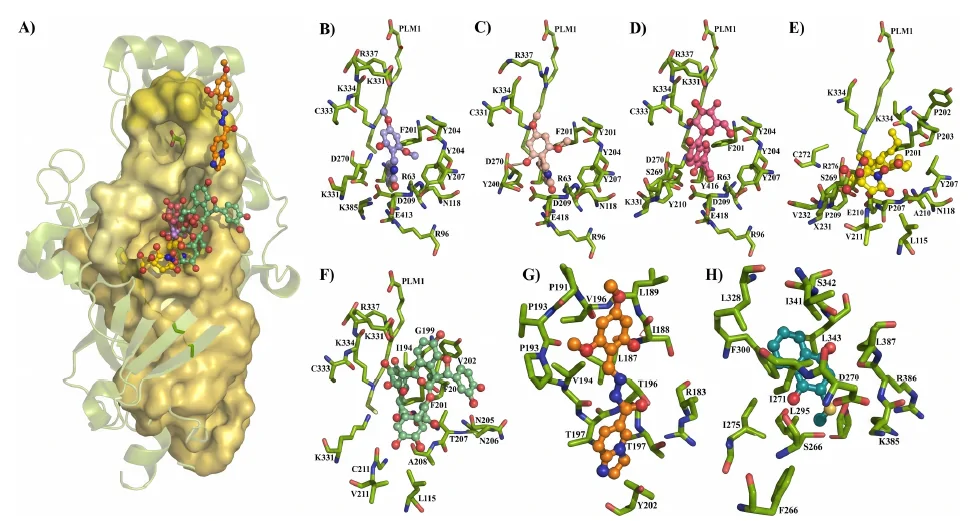
All assayed ligands docked on the occupied conformation of CD36 yielded by MOE software. (**A**) Overlapping of the binding modes from the assayed ligands depicting the interaction mostly with the SMAC, (**B**) Binding mode of AP5055, (**C**) Binding mode of AP5156, (**D**) Binding mode of Puerarin, (**E**) Binding mode of SSO, (**F**) Binding mode of Salvianolic acid, (**G**) Binding mode of AP5258 (**H**) Binding mode of MTN. H-bonds are depicted as red dashes.

**Fig. (5):**
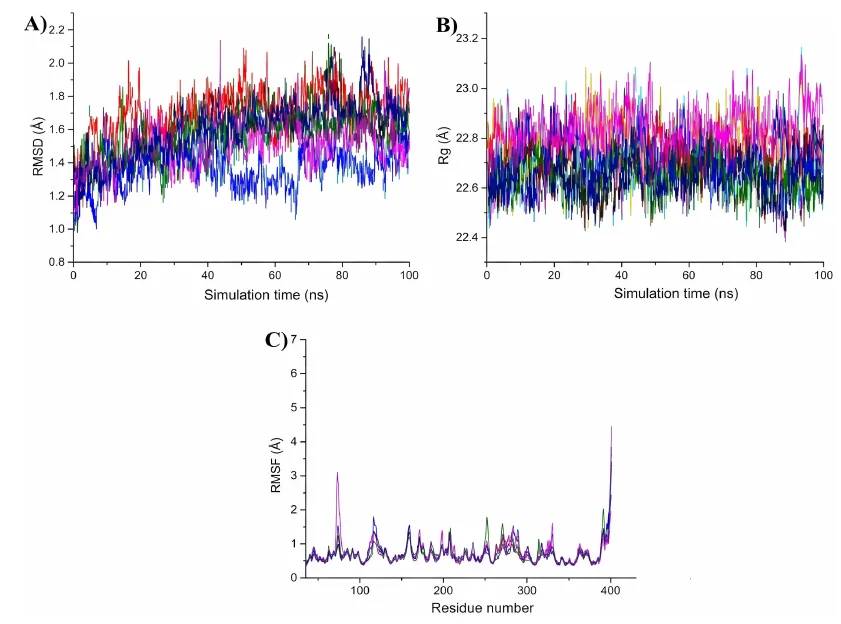
RMSD, Rg, and RMSF analysis of the ligand-receptor complexes in the unoccupied conformations of CD36. (**A**) RMSD, (**B**) Rg, and (**C**) RMSF. The receptor-ligand complexes are coloured as follows: receptor-AP5055 (black-line), receptor-AP5156 (red line), receptor-AP5258 (blue line), receptor-Puerarin (pink line), receptor-Salvianolic acid (green line), and receptor-SSO (dark blue line) complexes.

**Fig. (6):**
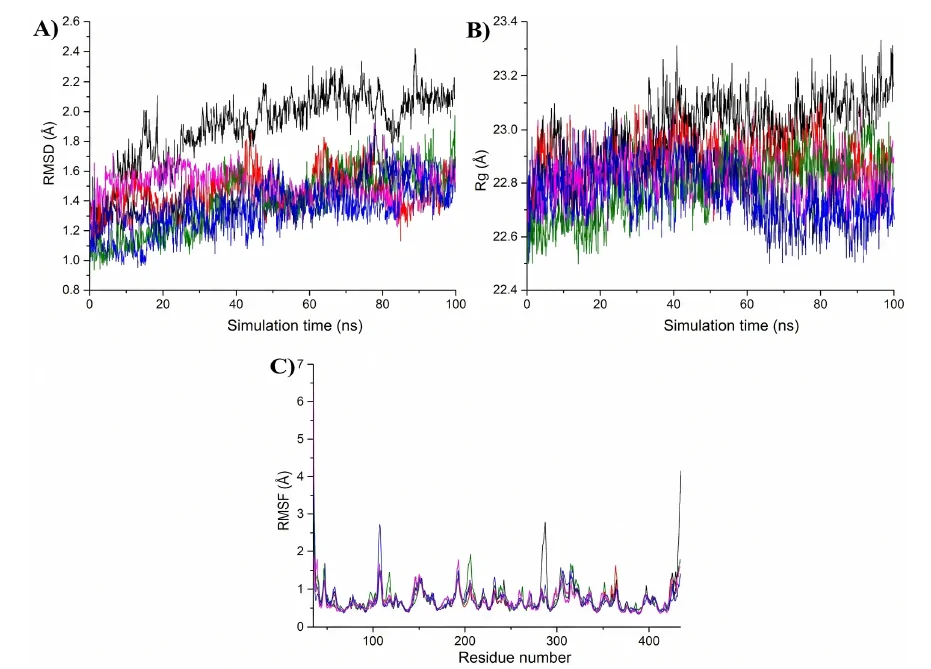
RMSD, Rg, and RMSF analysis of the ligand-receptor complexes in the occupied conformations of CD36. (**A**) RMSD, (**B**) Rg, and (**C**) RMSF. The receptor-ligand complexes are coloured as follows: receptor-AP5055 (black-line), receptor-AP5156 (red line), receptor-AP5258 (blue line), receptor-Puerarin (pink line), receptor-Salvianolic acid (green line), and receptor-SSO (dark blue line) complexes.

**Fig. (7):**
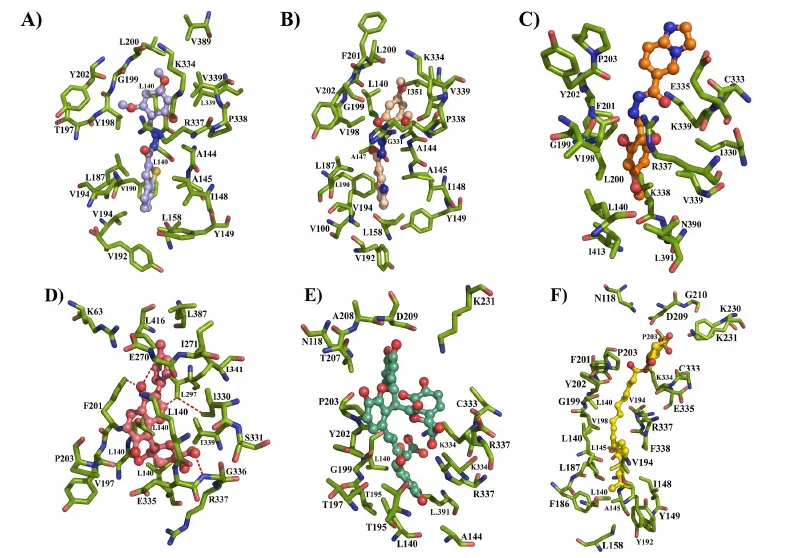
Binding modes from the MD simulations of the ligand-receptor complexes analyzed in the unoccupied conformation of CD36. (**A**) Binding mode of AP5055, (**B**) Binding mode of AP5156, (**C**) Binding mode of AP5258, (**D**) Binding mode of Puerarin, (**E**) Binding mode of Salvianolic acid, and (**F**) Binding mode of SSO. H-bonds are depicted as red dashes.

**Fig. (8):**
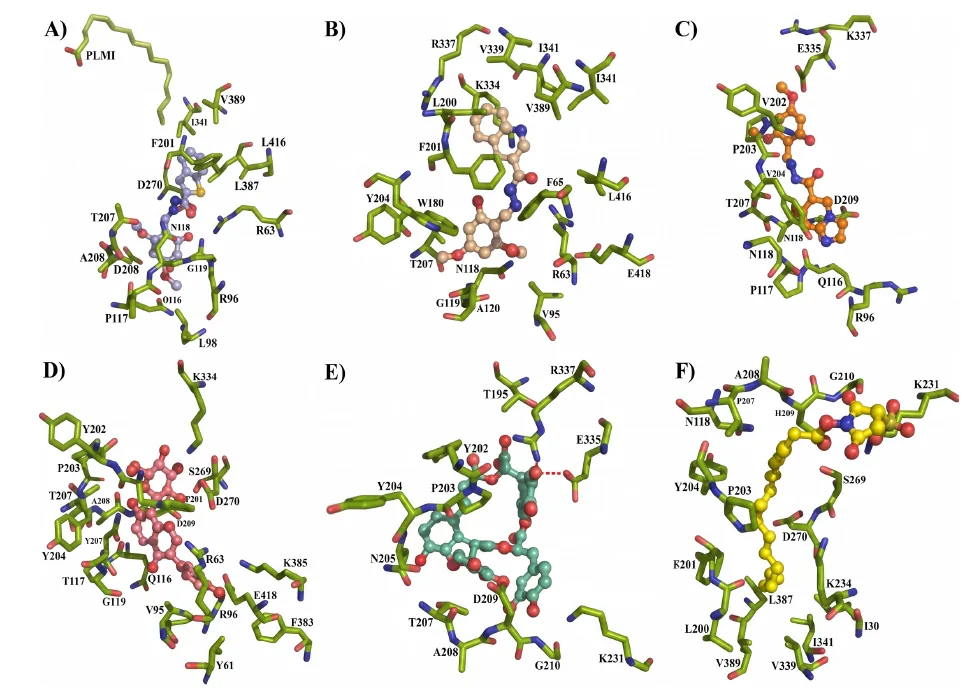
Binding modes from the MD simulations of the ligand-receptor complexes analyzed in the occupied conformation of CD36. (**A**) Binding mode of AP5055, (**B**) Binding mode of AP5156, (**C**) Binding mode of AP5258, (**D**) Binding mode of Puerarin, (**E**) Binding mode of Salvianolic acid, and (**F**) Binding mode of SSO. H-bonds are depicted as red dashes.

**Table 1:** Analysis of interacting residues in the unoccupied conformation of CD36 was performed using MOE.

**Ligand**	**Binding Energy (kcal/mol)**	**Non-bonded Interactions**
*AP5055*	-5.73	R63, R96, N118, F201, P203, Y204, T207, D209, K231, D270, C333, K334, E335, R337, K385, L416, E418
*AP5156*	-5.76	R63, R96, N118, F201, P203, Y204, T207, D209, S269, D270, C333, K334, R337, L416, E418
*AP5258*	-7.23	T197, V198, G199, L140, A141, A144, L200, F201, Y202, P203, Y204, K334, R337, V339, L391
*Puerarin*	-5.87	R63, R96, F201, P203, Y204, T207, D209, Y230, K231, S269, D270, C333, K334, E335, R337, L416, E418
*Salvianolic acid*	-6.95	L115, G119, L200, F201, Y202, P203, N205, N206, T207, A208, G210, V211, K231, C333, K334, E335, R337
*SSO*	-6.37	L115, N118, L200, F201, Y202, P203, T207, A208, D209, G210, V211, Y230, K231, G232, S269, D270, C272, K334
*MTN*	-5.41	F266, S268, T269, D270, I271, I275, L295, F300, L328, I341, S342, L343, K385, R386, L387

**Table 2:** Analysis of the interacting residues in the occupied conformation of CD36 was performed using MOE.

**Ligand**	**Binding Energy (kcal/mol)**	**Non-bonded Interactions**
*AP5055*	-5.73	R63, R96, N118, F201, P203, Y204, T207, D209, K231, D270, C333, K334, E335, R337, K385, L416, E418, PLM1
*AP5156*	-5.76	R63, R96, N118, F201, P203, Y204, T207, D209, S269, D270, C333, K334, R337, L416, E418, PLM1
*AP5258*	-5.32	R183, L187, S188, L189, V190, P191, Y192, P193, V194, T195, T196, T197, Y202
*Puerarin*	-5.86	R63, R96, F201, P203, Y204, T207, D209, Y230, K231, S269, D270, C333, K334, E335, R337, L416, E418, PLM1
*Salvianolic acid*	-6.97	L115, G199, L200, F201, Y202, P203, N205, N206, T207, A208, G210, V211, K231, C333, K334, E335, R337, PLM1
*SSO*	-6.36	L115, N118, L200, F201, Y202, P203, T207, A208, D209, G210, V211, Y230, K231, G232, S269, D270, C272, K334, PLM1
*MTN*	-5.39	F266, S268, D270, I271, I275, L295, F300, L328, I341, S342, L343, K385, R386, L387

**Table 3:** RMSD and RG values calculated during the equilibrium state for each receptor-ligand system (final 20 ns of the equilibrated trajectory). Values represent mean ± SD.

**CD36 conformation**	**Ligand**	**RMSD (Å) Mean ± SD**	**Rg (Å) Mean ± SD**
Unoccupied	*AP5055*	1.61 ± 0.08	22.60 ± 0.08
	*AP5156*	1.76 ± 0.10	22.76 ± 0.07
	*AP5258*	1.41 ± 0.09	22.63 ± 0.08
	*Puerarin*	1.57 ± 0.10	22.86 ± 0.08
	*Salvianolic acid*	1.64 ± 0.08	22.62 ± 0.06
	*SSO*	1.76 ± 0.12	22.72 ± 0.10
Occupied	*AP5055*	2.05 ± 0.10	23.10 ± 0.07
	*AP5156*	1.44 ± 0.10	22.86 ± 0.07
	*AP5258*	1.45 ± 0.09	22.69 ± 0.05
	*Puerarin*	1.54 ± 0.08	22.81 ± 0.07
	*Salvianolic acid*	1.61 ± 0.10	22.88 ± 0.08
	*SSO*	1.54 ± 0.11	22.68 ± 0.08

**Table 4:** Binding free energy components of ligand/receptor complexes analyzed in the occupied and unoccupied conformations of CD36, calculated using the MMGBSA approach (values in kcal/mol).

**CD36 Conformation**	**Ligand**	**ΔEvdw**	**ΔEele**	**ΔGele, sol**	**ΔGnpol, sol**	**ΔGcal**
Occupied	*AP5055*	-38.96 ± 1.0	-17.11 ± 5.8	38.55 ± 5.4	-5.37 ± 0.20	-22.91 ± 0.8
	*AP5156*	-32.06 ± 10.1	-11.96 ± 6.4	29.41 ± 12.7	-4.44 ± 1.40	-19.06 ± 6.9
	*AP5258*	-37.77 ± 6.8	-18.53 ± 3.4	37.45 ± 5.0	-4.40 ± 0.74	-23.26 ± 2.2
	*Puerarin*	-38.63 ± 3.7	-46.28 ± 2.8	66.61 ± 0.13	-5.78 ± 0.25	-24.09 ± 1.4
	*Salvianolic acid*	-25.34 ± 3.6	-35.98 ± 13.7	52.96 ± 18.7	-3.81 ± 0.91	-12.18 ± 2.5
	*SSO*	-35.67 ± 11.0	-38.29 ± 22.3	56.40 ± 27.9	-5.30 ± 1.65	-22.87 ± 7.2
Unoccupied	*AP5055*	-47.15 ± 0.8	-14.81 ± 1.6	29.76 ± 1.5	-6.42 ± 0.11	-38.63 ± 0.9
	*AP5156*	-49.66 ± 1.7	-9.38 ± 1.5	28.19 ± 2.0	-6.40 ± 0.29	-37.27 ± 2.1
	*AP5258*	-44.82 ± 3.2	-22.81 ± 1.3	40.99 ± 0.9	-5.73 ± 0.32	-32.38 ± 3.6
	*Puerarin*	-42.73 ± 12.7	-39.84 ± 12.0	63.24 ± 8.0	-6.06 ± 0.96	-25.40 ± 9.7
	*Salvianolic acid*	-48.58 ± 6.3	-154.04 ± 42.8	183.46 ± 38.0	-7.09 ± 0.64	-26.26 ± 10.5
	*SSO*	-58.03 ± 2.3	-68.92 ± 18.2	111.72 ± 15.9	-8.79 ± 0.29	-24.03 ± 1.05

**Table 5:** Interacting residues yielded by MD simulations from the MOE complexes.

**CD36 Conformation**	**Ligand**	**Non-bonded interactions**
Unoccupied	*AP5055*	L140, A141, A144, A145, I148, Y149, L158, F186, L187, V190, Y192, V194, T197, V198, G199, L200, Y202, K334, R337, P338, V339, V389, L391
	*AP5156*	L140, A141, A143, A144, A145, I148, Y149, L158, F186, L187, V190, Y192, V194, V198, G199, L200, F201, Y202, K334, R337, P338, V339, L391
	*AP5258*	L140, V198, G199, L200, F201, Y202, P203, I330, C333, K334, E335, R337, V339, V389, N390, L391, I413
	*Puerarin*	R63, L140, G199, L200, F201, Y202, P203, D270, I271, I330, S331, K334, E335, G336, R337, V339, I341, L387, V389, L416
	*Salvianolic acid*	N118, L140, A144, T195, T197, V198, G199, L200, Y202, P203, T207, A208, D209, K231, C333, K334, E335, R337, V339, L391
	*SSO*	N118, L140, A141, A144, A145, I148, Y149, L158, F186, L187, V190, Y192, V194, V198, G199, L200, F201, Y202, P203, D209, G210, Y230, K231, S269, C333, K334, E335, R337, P338, V339, L391
Occupied	*AP5055*	R63, R96, L98, Q116, P117, N118, G119, F201, T207, A208, D209, D270, I341, L387, V389, L416, PLM1
	*AP5156*	R63, F65, V95, N118, G119, A120, W180, L200, F201, Y204, T207, I330, K334, R337, V339, I341, V389, L416, E418
	*AP5258*	R96, Q116, P117, N118, Y202, P203, Y204, T207, A208, D209, E335, R337
	*Puerarin*	V61, R63, V95, R96, Q116, P117, N118, G119, F201, Y202, P203, Y204, T207, A208, D209, S269, D270, K334, F383, K385, E418
	*Salvianolic acid*	T195, Y202, P203, Y204, N205, T207, A208, D209, G210, K231, E335, R337
	*SSO*	N118, L200, F201, P203, Y204, T207, A208, D209, G210, K231, S269, D270, I330, K334, V339, I341, L387, V389

## Data Availability

The datasets used and/or analysed during the current study are available from the corresponding author on reasonable request.

## LIST OF ABBREVIATIONS

PTMs: Post-Translational Modifications
LCFAs: Long Chain Fatty Acids
FA: Fatty Acid
oxLDL: Oxidized Low-Density Lipoprotein
oxPC: Oxidized Phosphatidylcholine
MD: Molecular Dynamics
GAFF: General Amber force field
