# Supplementary material for: Unveiling the Small Molecules Binding Site of CD36 Cell Surface Receptor Through Docking and Molecular Dynamics Simulations
Source: Curr Med Chem. 2026 Feb 26;33(25):5339–59. doi: 10.2174/0109298673413172251202110617 (PMC13692764; doi:10.2174/0109298673413172251202110617)
Supplement: Supplementary file 1 [file CMC-33-25-5339_SD1.pdf]

Supplementary Material

Unveiling the Small Molecules Binding Site of CD36 Cell Surface Receptor Through Docking and Molecular Dynamics Simulations

Naomi Montes-Mondragón[1], Rosa Salgado-Brito[1], José-Rubén García-Sánchez[2], Martiniano Bello[3], José Correa-Basurto[3], Vicente Sandoval-Herrera[4], Sergio-Roberto Zamudio-Hernández[4] and David Méndez-Luna[3,4,*]

[1]Universidad Simón Bolívar, Av. Río Mixcoac Nº 48, Col. Insurgentes, Mixcoac, Benito Juárez, Ciudad de México C.P. 03920, México; [2]Laboratorio de Oncología Molecular y Estrés Oxidativo de la Escuela Superior de Medicina, Instituto Politécnico Nacional, Plan de San Luis y Díaz Mirón, s/n, Col. Casco de Santo Tomas, Ciudad de México, 11340, México; [3]Laboratorio de Diseño y Desarrollo de Nuevos Fármacos e Innovación Biotecnológica, Escuela Superior de Medicina, Instituto Politécnico Nacional, Plan de San Luis y Díaz Mirón s/n, Col. Casco de Santo Tomas, Alcaldía Miguel Hidalgo, C.P. 11340, Mexico City, México; [4]Departamento de Fisiología, Escuela Nacional de Ciencias Biológicas, Instituto Politécnico Nacional, Zacatenco, Av. Wilfrido Massieu 399, Col. Nueva Industrial Vallejo, Alcaldía Gustavo A. Madero, C.P. 07738, Ciudad de México, México

SEARCH FOR PROBABLE CD36 LIGANDS

A rational search was conducted using scientific databases including PubMed (https://pubmed.ncbi.nlm.nih.gov/), Scopus (https://www.scopus.com/search/form.uri?display=basic# basic), SciELO (https://scielo.org/es/), and Google Academics (https://scholar.google.es/schhp?hl=es). The keywords used were: *CD36 activity*, *CD36 ligands*, *docking*, and *MD simulations*. From the search results, only studies that did not involve *in silico* simulations (which could introduce bias into our analysis) were selected. Instead, preference was given to sources containing robust experimental biological data supporting CD36 modulation. This approach identified three derivatives from N'-(2-hydroxy-4,6-dimethoxybenzylidene)acetohydrazide scaffold (AP5055, AP5156, and AP5258), two polyphenolic compounds (Puerarin and Salvianolic acid), and two well-known inhibitors of this receptor (SSO and MTN) as potential ligands for CD36.

MOLECULAR DOCKING PROTOCOL WITH ADT SOFTWARE

The interaction between the analyzed ligands and CD36 was evaluated using AutoDock (v4.2.6) as a complementary approach. In detail, polar hydrogen atoms were added to the CD36 residues capable of forming polar contacts, and Kollman charges were calculated. For ligands, Gasteiger charges were assigned. A grid-based procedure was delimited to generate the affinity maps setting a blind docking procedure covering almost the entire ectodomain of the receptor. As scoring sampling was employed the Lamarckian genetic algorithm, establishing the following search parameters: a randomized initial population of 100 individuals, elitism set to a single survivor *per* cycle, and a maximum of $1x10^7$ energy evaluations.

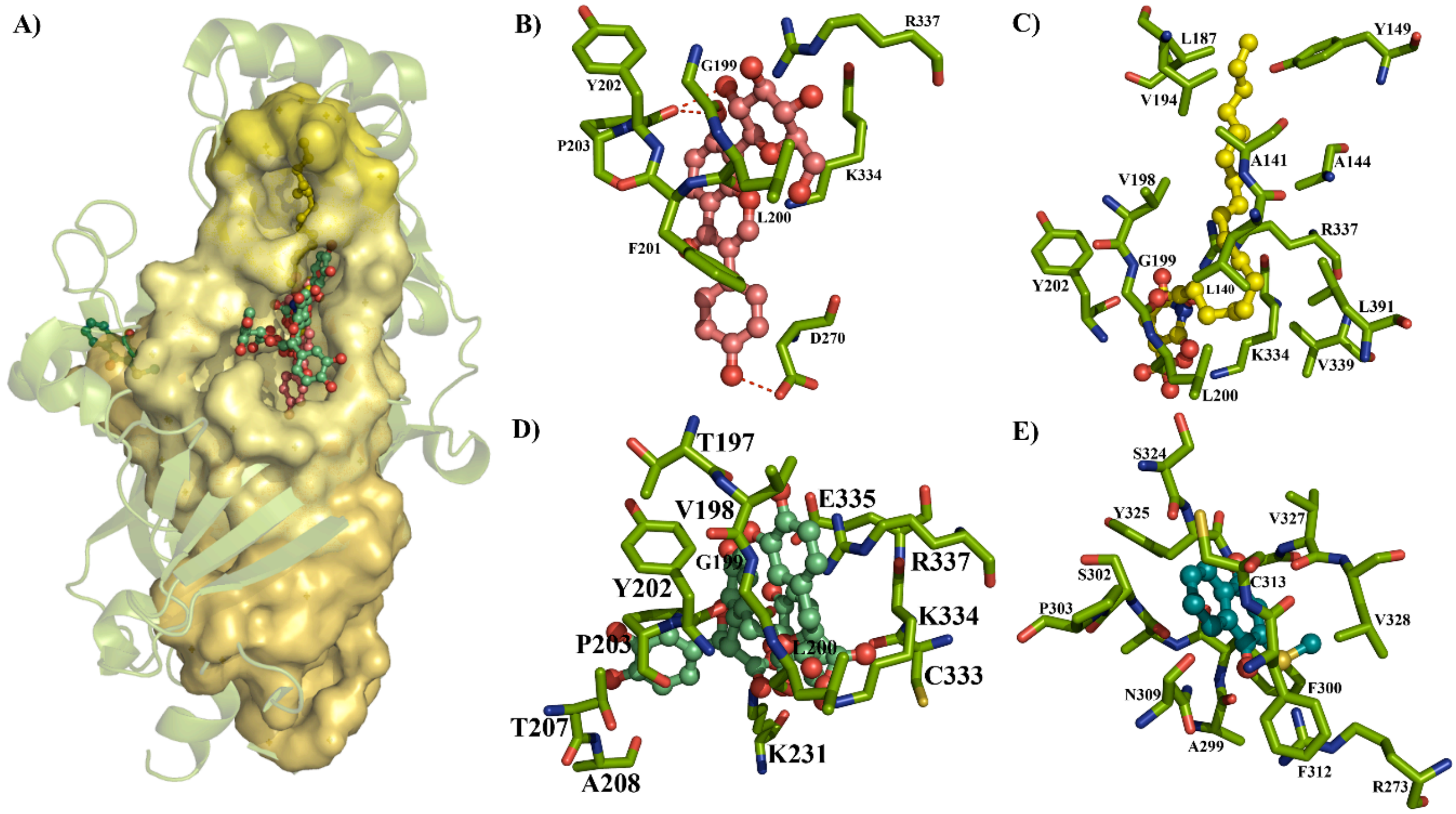

Fig. (S1). Puerarin, SSO, Salvianolic acid and MTN docked on the unoccupied conformation of CD36 yielded by ADT software. (**A**) Overlapping of the bindings modes from the assayed ligands depicting mostly the interaction with the SMAC, (**B**) Binding mode of Puerarin, (**C**) Binding mode of SSO, (**D**) Binding mode of Salvianolic acid, and (**E**) Binding mode of MTN. H-bonds are depicted as red dashes.

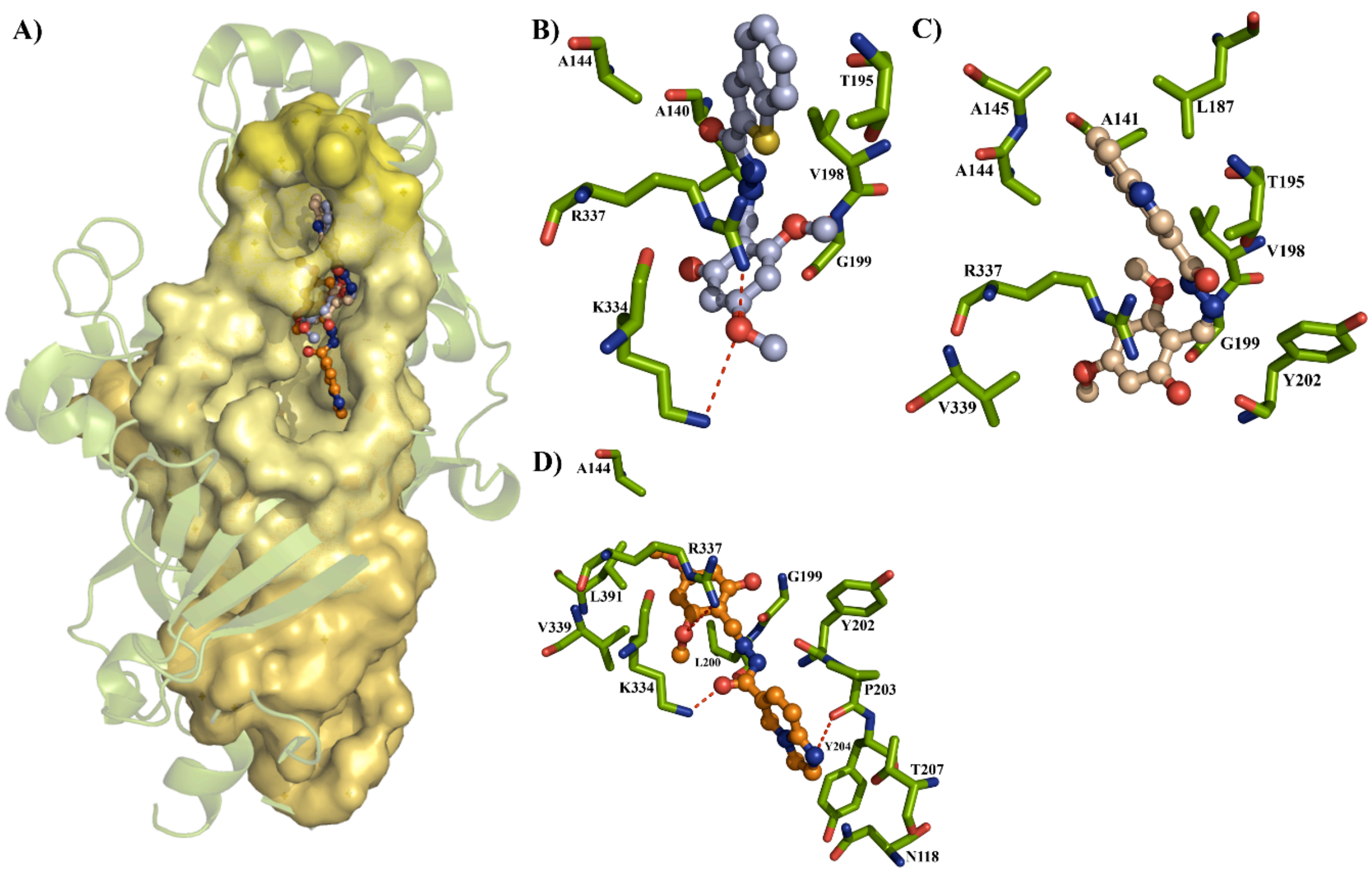

Fig. (S2). AP5055, AP5156 and AP5258 docked on the unoccupied conformation of CD36 yielded by ADT software. (**A**) Overlapping of the bindings modes from the assayed ligands depicting the interaction with the SMAC, (**B**) Binding mode of AP5055, (**C**) Binding mode of AP5156, and (**D**) Binding mode of AP5258.

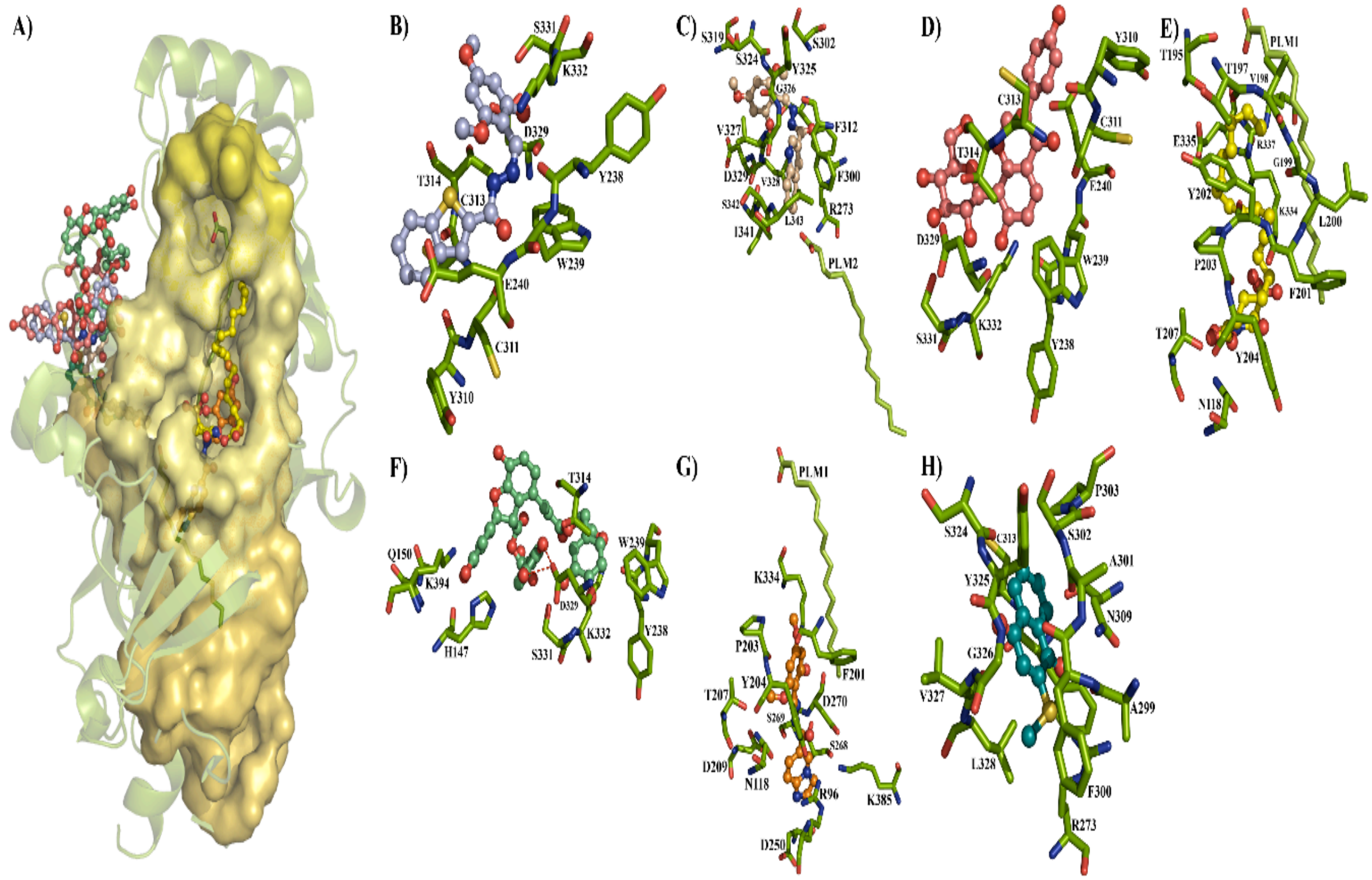

Fig. (S3). All assayed ligands docked on the occupied conformation of CD36 yielded by ADT software. (**A**) Overlapping of the bindings modes from the assayed ligands, (**B**) Binding mode of AP5055, (**C**) Binding mode of AP5156, (**D**) Binding mode of Puerarin, (**E**) Binding mode of SSO, (**F**) Binding mode of SSO, (**F**) Binding mode of Salvianolic acid, (**G**) Binding mode of AP5258, and (**H**) Binding mode of MTN. H-bonds are depicted as red dashes.

Table S1. Analysis of the interacting residues in the unoccupied conformation of CD36 yielded by ADT.

Ligand	Binding Energy (kcal/mol)	Non-bonded Interactions
AP5055	-8.31	L140, A144, T195, V198, G199, K334, R337
AP5156	-8.26	A141, A144, A145, L187, T195, V198, G199, Y202, R337, V339
AP5258	-8.71	N118, A144, G199, L200, Y202, P203, Y204, T207, K334, R337, V339, L391
Puerarin	-8.31	G199, L200, F201, Y202, P203, D270, K334, R337
Salvianolic acid	-6.69	T197, V198, G199, L200, Y202, P203, T207, A208, K231, E235, C333, K334, R337
SSO	-7.99	L140, A141, A144, Y149, L187, V194, V198, G199, L200, Y202, K334, R337, V339, L391
MTN	-7.58	R273, A299, F300, S302, P303, N309, F312, C313, S324, Y325, V327, V328

Table S2. Analysis of the interacting residues in the occupied conformation of CD36 yielded by ADT.

Ligand	Binding Energy (kcal/mol)	Non-bonded Interactions
AP5055	-6.99	Y238, W239, E240, Y310, C311, C313, T314, D329, S331, K332
AP5156	-10.8	R273, F300, S302, F312, S319, S324, Y325, G326, V327, L328, D329, I341, S342, L343, PLM2
AP5258	-6.7	R96, N118, F201, P203, Y204, T207, D209, D250, S268, S269, D270, K334, K385, PLM1
Puerarin	-5.76	Y238, W239, E240, Y310, C311, C313, T314, D329, S331, K332
Salvianolic acid	-6.59	H147, Q150, Y238, W239, T314, D329, S331, K332, K394
SSO	-7.65	N118, T195, T197, V198, G199, L200, F201, Y202, P203, Y204, T207, K334, E335, R337, PLM1
MTN	-7.69	R273, A299, F300, A301, S302, P303, N309, C313, S324, Y325, G326, V327, L328
